# Antioxidant activities of essential oil of *Bidens pilosa* (Linn. Var. Radita) used for the preservation of food qualities in North Cameroon

**DOI:** 10.1002/fsn3.330

**Published:** 2016-01-18

**Authors:** Augustin Goudoum, Armand B. Abdou, Léonard Simon T. Ngamo, Martin Benoît Ngassoum, Carl M. F. Mbofung

**Affiliations:** ^1^ Department of AgricultureLivestock and Derived ProductsThe University of MarouaThe Higher Institute of SahelP.O. Box 46MarouaCameroon; ^2^ Department of Biological SciencesThe University of NgaoundereP.O. Box 454NgaoundereCameroon; ^3^ Department of Applied ChemistryNational High School of Agro Industrial SciencesThe University of NgaoundereP. O. Box 455NgaoundereCameroon; ^4^ Departments of Food and Bioresource TechnologySchool of TechnologyThe University of BamendaP.O. Box 39BambiliCameroon

**Keywords:** Antioxidant activity, *Bidens pilosa*, Essential oil, storage condition

## Abstract

This study aimed to determine the total antioxidant capacity of the essential oil (EO) of leaves of *Bidens pilosa* (Linn. Var. Radita**)** used as protectant of stored grains in Northern Cameroon. EO was characterized by GC‐FID, antioxidant activity (AA) was determined by combining: evaluation of radical‐scavenging activity, reducing power (RP) and co‐oxidation of *β*‐carotene methods. Tests were carried out on crude and stored EO kept for two weeks at 31.48 ± 2.88°C and 58.56 ± 6.78% relative humidity. These conditions are the same as those of grain storage. GC analyses enabled the identification of 27 compounds, representing around 97.57% of the total oil contents. The major constituents of the EO were *α*‐pinene (14.7%), *ε*‐caryophyllene (13.5), and *β*‐ocimene (12.8%). The AA of the crude and stored EO are proportional to the concentrations and time of exposition. Exposed at the day light, this EO inhibit 77.4–18.69% for the DPPH system, 59.55–19.14% for RP method and 91.88–21.8% for *β*‐carotene‐linoleate model system, respectively, from crude and 15 days storage EO at 20 mg L^−1^. For the EC_50_ values, *β*‐carotene method is excellent and in the decreasing order of DPPH method, PR with 2.52 mg L^−1^, 2.77 mg L^−1^ and 4.13 mg L^−1^, respectively, for the crude oil. The ET_50_ were 1.59 days for the RP method and 2.88 days DPPH system and *β*‐carotene‐linoleate model system at 20 mg L^−1^. These results showed that the EO of *B. pilosa* leaves exhibits AA that might be an added value for this EO preventing stored products from pest attacks.

## Introduction

The quality of the food, in its different components sanitary, nutritional, and hedonic, is a major preoccupation for consumers in industrial agroalimentary and professionals of health sectors (CIRAD 2007). The food industry is currently facing tremendous pressure from consumers because of the use of chemical protectants to prevent noxious activities of food‐borne microbes (Deba et al. 2008). To answer these preoccupations and provide a better appreciation tool to operators at each level of the process, including the producers, it is necessary to adapt the fresh and transformed products to the demand of markets and consumers needs. This may help to preserve the biodiversity, though the reduction of the use of chemicals as tools to reduce post‐harvest losses (CIRAD 2007). A new approach to prevent food from insect and microorganism damage or protect food from oxidation, an essential oil is used as a preservative (Deba et al. 2008; Goudoum et al. 2009, 2013).

In Northern Cameroon, peasants usually introduce local aromatic plants in their granaries while filling them. This practice aimed to protect stored grains against the insect pest attacks (Anonyme 2004). These plants are also used in the traditional pharmacopoeia to cure infection. Recent work on natural products drifted from the aromatic plants because of their biodegradability, their anti‐insect efficiency and their weak persistence; as alternative to the conventional insecticides for the control of the stored grain pests. The secondary metabolites of aromatic plants are known for their insecticide activities against different species of insects (Huang et al. 2000). The use of these essential oils on food for conservation adds flavor as well(Goudoum et al. 2009). However, these essential oils contain bioactive compounds that play diverse biological roles. In addition to their anti‐insect potential, they exhibit other biological activities as their antioxidant potential is capable to prevent the formation of free radicals by oxidization of fatty acids within food (Goudoum et al. 2009).

In this respect, essential oils of some aromatic plants of Northern Cameroon are described to have effectiveness in the control of stored grain insect pests (Taponjou et al. 2002; Ngamo et al. 2007; Goudoum et al. 2009). *Bidens pilosa* (Linn. Var. Radita**)** is one of the cited insecticidal plants to protect food against insect attacks in the Far‐North of Cameroon. *B. pilosa* is an annual, erect, and ruderal herb originating from South America and now found in almost all tropical and subtropical region countries (Geissberger and Séquin 1991; Grombone‐Guaratini et al. 2005). Nevertheless, this plant is also commonly used in traditional medicine. The first report on the essential oils composition, antioxidant, antibacterial, and antifungal activities of *B. pilosa* leaves and flower from Japan was done by Deba et al. (2008). The plant is used in various folk medicines such as anti‐inflammatory, antiseptic, liver‐protective, blood pressure lowering, hypoglycemic effects (Dimo et al. 2002) and biological activities against storage insects and microorganism, and as an antioxidant (Deba et al. 2008). Previous phytochemical studies on this plant have proved the occurrence of flavonoids, polysaccharides, carotenoids, amines, lactones, mineral elements, coumarins, and volatile oil. These compounds were used because of its antioxidant potential (Chiang et al. 2004; Tomczykowa et al. 2011). The plant composition and antioxidant activities of the Genus *Bidens* were assayed in Japan, Poland, Taiwan , and as a traditional medicine to prevent inflammation and cancer (Deba et al. 2008; Tomczykowa et al. 2011). In Cameroon, the study done by Zollo et al. (1995) were focused on the chemical composition of South region. Therefore, the aim of the present study was carried out because of antioxidant activities of *B. pilosa* and the essential oil of *B. pilosa* leaves in Northern Cameroon were used to prevent storage grains against insects and microorganism.

## Material and Methods

### Plant collection and extraction of essential oils

Fresh leaves of *B. pilosa* were collected from Moutourwa in Far‐Nord Cameroon in June 2015. The Department of Diamare is located in the far north region between 10° and 11° north latitude and 14° and 15°east.

Fresh leaves were collected and dried in the shade for 24 h and cut into pieces. Once dried, 1 kg of leaves of *B. pilosa* was hydrodistillated in a Clevenger‐type apparatus for 4 h as described by Goudoum et al. (2009). The distillated oil was preserved in sealed sample tubes and stored in a refrigerator for analysis.

The crude essential oil of *B. pilosa* leaves was used directly for analyzing antioxidant activities in methanol to a concentration ranging from 1 to 20 mg L^−1^. A quantity of 2 mL for each concentration of essential oil was exposed during 5, 10, and 15 days in similar conditions used for those of grain storage: the temperature of 31.48 ± 2.88°C and 58.56 ± 6.78% relative humidity. These 15 days corresponded to the delay of persistence of insecticidal activity of this essential oil on stored grains. As control an amount of 0.1–2 mg L^−1^of Butylated Hydroxytoluene (BHT) was used.

### Chemical components

Linoleic acid, *β*‐carotene and 1,1‐diphenyl‐2‐picrylhydrazyl (DPPH), BHT, Potassium ferricyanide, nitro blue tetrazolium (NBT), trichloroacetic acid (TCA), and ferric chloride were purchased from Prolabo. All other reagents were of analytical grade.

### Analysis of chemical composition of essential oils

The GC/FID (Chromatograph Agilent HP‐6820) was carried out with HP‐5MS column (5% phenyl methyl siloxane) with 30 m length and 250 *μ*m in diameter and 1 *μ*m of thickness. The carrier gas was hydrogen, the oven temperature was programmed from 40 to 230°C with a rate of 5°C min^−1^ with a stay at 230°C during 5 min. The pressure of the carrier gas was 49.9 KPa and the flux at 74.1 mL min^−1^. Quantification was carried out by percentage of peak area calculation. The identification of single compounds was performed by comparison of the retention indices with reference data (Davies 1990; Kouroussou et al. 1998).

### DPPH radical‐scavenging activity

The antioxidant activity of the essential oils based on the scavenging activity of the stable 1,1‐diphenyl‐2‐ picrylhydrazyl free radical was determined by the method described by Braca et al. (2001). Different concentrations of essential oil (1, 5, 10, 15, and 20 mg L^−1^) were added to 3 mL of a 0.004% MeOH solution of DPPH. Water (0.1 mL) in place of the essential oil was used as control. BHT was used for comparison. Absorbance at 517 nm was determined after 30 min, using a PharmaSpec MODEL UV‐1700 spectrophotometer, and the percent inhibition activity was calculated as:

[(A0−A1)/A0]·100;

where A0 was the absorbance of the control, and A1 was the absorbance of the extract/standard.

### Reducing power

The reducing power was determined according to the method of Oyaizu (1986). Each concentration of essential oil (1, 5, 10, 15, and 20 mg L^−1^) in methanol (2.5 mL) was mixed with 2.5 mL of 200 mmol L^−1^ sodium phosphate buffer (pH 6.6) and 2.5 mL of 1% potassium ferricyanide, and the mixture was incubated at 50°C for 20 min. After 2.5 mL of 10% trichloroacetic acid (w/v) was added. Two millilitres of mixture was added to 5 mL of deionized water and 100 *μ*L of 0.1% ferric chloride, and the absorbance was measured at 700 nm against a blank in a PharmaSpec MODEL UV‐1700 spectrophotometer. A higher absorbance indicates a higher reducing power. BHT was used for comparison.

### Antioxidant assay using a *β*‐carotene‐linoleate model system

The co‐oxidation of *β*‐carotene of essential oil was evaluated by the *β*‐carotene‐linoleate model system (Miller 1971). A solution of *β*‐carotene was prepared by dissolving 2 mg of *β*‐carotene in 10 mL of chloroform. Two milliliters of this solution was introduced into a 100 mL round bottom flask. After chloroform was removed under vacuum, 40 *μ*L of purified linoleic acid, 400 mg of Tween 40 emulsifier, and 100 mL of aerated distilled water were added to the flask with vigorous shaking. Aliquots (4.8 mL) of this emulsion were transferred into different test tubes containing different concentrations (1, 5, 10, 15 and 20 mg L^−1^) of the essential oils (0.2 mL). As soon as the emulsion was added to each tube, the zero time absorbance was measured at 470 nm, using a PharmaSpec MODEL UV‐1700 spectrophotometer. The tube was placed at 50°C in a water bath and absorbance was recorded and measured after 2 h; without *β*‐carotene (blank) was prepared for background subtraction. The same procedure was repeated with the synthetic antioxidant BHT, as positive control. Antioxidant activity was calculated, using the following equation:

$$\begin{array}{cc} & \text{Antioxidant activity}\;=\;(\beta -\text{carotene content after}\\ & \;\;\text{2 h of assay}/\text{initial}\;\beta -\text{carotene content})\;\times \;100. \end{array}$$

### Statistical analysis

Experimental results were expressed as means with standard deviation. The data were analyzed by an analysis of variance (*P* < 0.05) and the means separated by Duncan's multiple range test with XLSTAT 2014 software (add‐on for Microsoft Excel^®^ of Microsoft Corporation, USA). The effective concentration at which the 50% of antioxidant activities of essential oil was inhibited (EC_50_) and the effective time at which 50% of antioxidant activities of essential oil was inhibited (ET_50_) was calculated from linear regression analysis, using PROINRA 2.0 (PROINRA S.A., Spain) software.

## Results and Discussion

### Chemical composition of essential oil of *Bidens pilosa*

The result of the chemical analysis of the essential oil of *B. pilosa* is represented in the Table 1. The yields of leaves oil obtained from the hydrodistillation procedures, calculated on a dry weight was 0.19% (v/w). GC‐FID analyses enabled the identification of 27 compounds, accounting for 97.57% of the total oil contents. The major oil constituents of the leaves were *α*‐pinene (14.7%), *ε*‐caryophyllene (13.5), *β*‐ocimene (12.8%), and cadinene (10.1%). The other significant compounds included Megastigmatrienone (7.1%), Cubebene (4.8%), linalool (4.4%), Bourbonene (3.9%), Caryophyllene oxide (3.5%), p‐cymene‐8‐ol (3.6), limonene (2.3%), and *α*‐terpinolene and Δ‐elemene (2.5%). The minor constituents were 1‐hexanol, *β*‐myrcene, *δ*‐3‐carene, *γ*‐Terpinene, trans‐linalool oxide, cis linalool oxide, terpinene‐4‐ol, Farnesene, isoledene, Methyleugenol, cis‐*β*‐Elemene, a‐Humulene, *β*‐Selinene, and *ε*‐nerolidol. Interestingly, the major constituents of the present results, *β*‐caryophyllene, cadinene, *α*‐pinene, limonene, *β*‐transocimene, *β*‐cis‐ocimene, *τ*‐muurolene, *β*‐bourbonene, *β* ‐elemene, *β* ‐cubebene, *ε*‐caryophyllene, caryophyllene oxide, and megastigmatrienone were previously reported to be of significant quantities of essential oils in the leaves of *B. pilosa* (Deba et al. 2008; Tomczykowa et al. 2011).

**Table 1:** Chemical composition obtained by GC‐FID of the crude essential oils of *Bidens pilosa* leaves collected in the Far‐North of Cameroon in June 2015

	RI^a^	Compounds	%
1	851	1‐hexanol	1.1
2	978	*α*‐pinene	14.7
3	995	*β*‐myrcene	1.9
4	1006	δ‐3‐carene	1.3
5	1026	*β*‐ocimene	12.8
6	1028	limonene	2.3
7	1029	*ρ*‐cymene‐8‐ol	3.6
8	1050	*α*‐terpinolene	2.5
9	1052	*γ*‐Terpinene	0.1
10	1079	trans‐linalool oxide	1.6
11	1091	Cis‐linalool oxide	1.4
12	1100	linalool	4.4
13	1146	terpinene‐4‐ol	0.3
14	1336	Bourbonene	3.9
15	1348	Δ‐elemene	2.5
16	1358	Farnesene	1.5
17	1366	isoledene	1.2
18	1372	Methyleugenol	0.1
19	1389	cis‐ *β*‐Elemene	0.1
20	1450	*ε*‐caryophyllene	13.5
21	1471	*α*‐Humulene	0.2
22	1491	*β*‐Selinene	0.1
23	1560	Megastigmatrienone	7.1
24	1565	*ε*‐nerolidol	1.2
25	1588	Caryophyllene oxide	3.5
26	1648	Cubebene	4.8
27	1658	Cadinene	10.1
Total			97.6

RI, Retention index.

a The compounds presented in this table are those having a proportion higher or equal than 0.1%.

According to the study carried out by Silva et al. (2011), the content of essential oil from flowers, leaves, and stems of *B. pilosa* has been analyzed by GCMS in China, Japan, USA, Cameroon, Nigeria, and Iran (Sakuda 1988; Zollo et al. 1995; Qin et al. 2003; Dong et al. 2004; Deba et al. 2008; Priestap et al. 2008; Riahi et al. 2008; Ogunbinu et al. 2009). In this review, the series of components identified as being commonly found in plants containing essential oil and present mostly in very small quantities are not listed. In the species, a series of mono‐ and sesquiterpenes have been detected (Zollo et al. 1995; Qin et al. 2003; Dong et al. 2004; Priestap et al. 2008; Riahi et al. 2008; Ogunbinu et al. 2009; Silva et al. 2011).

### Scavenging ability on 1,1‐diphenyl‐2‐picrylhydrazyl radical DPPH

The studied essential oil perfectly inhibits the free radical scavenging measured by DPPH assay as shown in Table 2. The activities of the crude and storage essential oil are proportional to the concentrations and time of exposition. The crude (1st day) essential oil of *B. pilosa* leaves inhibit 10.86–77.40%, respectively, at the concentration of 1 mg L^−1^ and 20 mg L^−1^. At 1–20 mg L^−1^, the scavenging abilities of essential oil exposed at light day increased to 8.07–53.87%; 3.26–38.90% and 2.38–28.69%, respectively, after 5, 10, and 15 days. A significant difference (*P* < 0.05) was observed between used essential oil concentration (*P* < 0.001) and exposure time (*P* < 0.001).

**Table 2:** Antiradical activity of essential oil of *Bidens pilosa* leaves observed with 1‐diphenyl‐2‐picrylhydrazyl during two weeks

Concentration (mg L^−1^)	Days				BHT^a^
	1	5	10	15	
1	10.86 ± 0.54^e1^	8.07 ± 0.68^d1^	3.26 ± 0.54^e2^	2.38 ± 0.65^d2^	47.27 ± 1.41^a^
5	48.44 ± 1.65^d1^	20.16 ± 1.59^c2^	11.41 ± 0.64^d3^	5.06 ± 0.31 ^cd1^	51.54 ± 0.95^b^
10	63.06 ± 0.12^c1^	48.52 ± 1.59^b2^	22.15 ± 1.13^c3^	6.19 ± 0.49^c4^	61.56 ± 1.26^c^
15	67.25 ± 1.69^b1^	52.35 ± 1.29^ab2^	28.9 ± 1.01^b3^	13.62 ± 1.73^b4^	69.6 ± 0.92^d^
20	77.4 ± 0.82^a1^	53.87 ± 2.08^a2^	34.56 ± 0.49^a3^	18.69 ± 1.52^a1^	84.24 ± 1.51^e^

a Butylated Hydroxytoluene concentration corresponds to 1/10 of the essential oil.

Averages followed by the same letter in the same column are not different significantly with *P* < 0.05 (Test of Duncan).

Averages followed by the same number in the same line are not different significantly with *P < 0.05* (Test of Duncan).

The DPPH method is based on the reduction of methanolic DPPH solution in the presence of a hydrogen‐donating antioxidant due to the formation of the nonradical form DPPH‐H by the reaction (Kumaran and karunakaran 2007). The essential oil studied was able to reduce the stable radical DPPH to the yellow‐colored diphenylpicrylhydrazine. It has been found that cysteine, glutathione, ascorbic acid, tocopherol, polyhydroxy aromatic compounds, and aromatic amines, reduce and decolorize 1,1‐diphenyl‐2‐picrylhydrazyl by their hydrogen‐donating ability (Blois 1958). It appears that the essential oil possesses hydrogen‐donating capabilities and acts as an antioxidant. The study by Goudoum et al. (2009)indicated that the essential oils of *Clausena anista* and *Plectranthus glandulosus* used to protect storage grains in Northern Region of Cameroon have a high antioxidant activity. The scavenging effect increased with increasing concentration of the essential oils. However, scavenging activity of BHT, a known antioxidant, was used as positive control, and has the higher activity than essential oils.

### Reducing power determination

Reducing powers of the essential oil increased with concentrations (Table 3). At 1%, the reducing powers of essential oil were 14.88, 7.77, 4.93, and 1.78 for crude, 5 days, 10 days, and 15 days exposed oil, respectively. For another concentration, the reducing powers of essential oil were in the range of 32.04–4.45, 42.04–10.93, 44–14.28, and 59.55–19.14%, respectively, for 5%, 10%, 15%, and 15%. However, reducing powers of BHT were 36.67 at 1% and 69.6 at 20%. A significant difference (*P* < 0.05) was observed between all essential oil concentration (*P* < 0.001) and exposure time (*P* < 0.001).

**Table 3:** Reducing power of essential oil of *Bidens pilosa* observed during two weeks

Concentration (mg L^−1^)	Days				BHT^a^
	1	5	10	15	
1	14.88 ± 0.52^e1^	7.77 ± 0.73^e2^	4.93 ± 0.73^e3^	1.78 ± 0.52^d4^	36.67 ± 1.10^d^
5	32.04 ± 0.15^d1^	25.04 ± 1.15^d2^	14.07 ± 1.39^d3^	4.45 ± 0.80^d4^	37.99 ± 0.22^d^
10	42.04 ± 1.18^c1^	32.78 ± 1.09^c2^	19.15 ± 1.27^c3^	10.93 ± 0.86^c4^	40.77 ± 0.65^c^
15	44 ± 0.45^b1^	36.72 ± 0.74^b2^	26.83 ± 1.15^b3^	14.28 ± 1.47^b4^	46.86 ± 0.49^b^
20	59.55 ± 0.98^a1^	41.65 ± 0.31^a2^	32.06 ± 0.81^a3^	19.14 ± 1.27^a4^	69.6 ± 0.79^a^

a Butylated Hydroxytoluene concentration corresponds to 1/10 of the essential oil.

Averages followed by the same letter in the same column are not different significantly with *P < 0.05* (Test of Duncan).

Averages followed by the same number in the same line are not different significantly with *P < 0.05* (Test of Duncan).

Previous works (Tanaka et al. 1988; Pin‐Der‐Duh 1998; Pin‐Der‐Duh et al. 1999) pointed out direct correlation between antioxidant activities and reducing power of certain plant extracts. The reducing properties are generally associated with the presence of reductones (Pin‐Der‐Duh 1998; Kumaran and karunakaran 2007), which have been shown to exert antioxidant action by breaking the free radical chain by donating an hydrogen atom (Gordon 1990). Reductions are also reported to react with certain precursors of peroxide, thus preventing peroxide formation (Kumaran and karunakaran 2007). Our data on the reducing power of essential oil suggest that it is likely to contribute significantly toward the observed antioxidant effect. However, the antioxidant activity of antioxidants have been attributed to various mechanisms, among which are prevention of chain initiation, binding of transition metal ion catalysts, decomposition of peroxides, prevention of continued hydrogen abstraction, reductive capacity, and radical scavenging (Kumaran and karunakaran 2007). Like the antioxidant activity, the reducing power of essential oils increased with increasing amount of sample (Goudoum et al. 2009). Nevertheless, the reducing power of BHT was more pronounced than that of essential oils.

### Antioxidant assay using a *β*‐carotene‐linoleate model system

In the present study, the essential oil was found to hinder the extent of *β*‐carotene bleaching by neutralizing the linoleate‐free radical and other free radicals formed in the system. The addition of essential oil at various concentrations prevented the bleaching of *β*‐carotene at different levels (Table 4). At 1%, the bleaching events were 16.45, 14.21, 10.17, and 4.08 for crude, 5 days, 10 days and 15 days exposed oil, respectively. This bleaching prevention was in the range of 38.09–8.39, 68.76–12.72, 81.01–19.23, and 91.88–21.80%, respectively, at 5%, 10%, 15%, and 15%. In comparison, the essential oil showed an appreciable antioxidant activity of 91.88 at 20%, while BHT, a synthetic antioxidant had 98.7% antioxidant activity at a concentration less than those of the oil. A significant difference (*P* < 0.05) was observed between all essential oil concentration (*P* < 0.001) and exposure time (*P* < 0.001).

**Table 4:** Antioxidant activity of essential oil of *Bidens pilosa* leaves observed during two weeks in *β*‐carotene‐linoleate system

Concentration (mg L^−1^)	Days				BHT^a^
	1	5	10	15	
1	16.45 ± 0.66^e1^	14.21 ± 0.89^e2^	10.17 ± 0.86^e3^	4.08 ± 0.33^e4^	23.13 ± 0.57^e^
5	38.09 ± 0.63^d1^	33.95 ± 0.28^d2^	18.75 ± 0.98^d3^	8.39 ± 0.67^d4^	44.18 ± 1.17^d^
10	68.76 ± 0.31^c1^	54.72 ± 0.31^c2^	27.04 ± 1.09^c3^	12.72 ± 0.21^c4^	77.36 ± 0.21^c^
15	81.01 ± 0.43^b1^	65.64 ± 0.71^b2^	37.16 ± 0.97^b3^	19.23 ± 0.86^b4^	94.13 ± 0.93^b^
20	91.88 ± 0.80^a1^	73.08 ± 0.64^a2^	49.05 ± 1.14^a3^	21.8 ± 0.78^a4^	98.7 ± 0.35^a^

a Butylated Hydroxytoluene concentration corresponds to 1/10 of the essential oil.

Averages followed by the same letter in the same column are not different significantly with *P* < 0.05 (Test of Duncan).

Averages followed by the same number in the same line are not different significantly with *P* < 0.05 (Test of Duncan).

As a result, *β*‐carotene is oxidized and broken down in part; subsequently, the system loses its chromophore and characteristic orange color, which can be monitored spectrophotometrically (Kumaran and karunakaran 2007). The presence of different antioxidants can hinder the extent of *β*‐carotene bleaching by neutralizing the linoleate‐free radical and other free radicals formed in the system (Jayaprakasha et al. 2001). Previous study done by Goudoum et al. (2009) shows that essential oils of *C. anisata* and *P. glandulosus* was found to hinder the extent of *β*‐carotene bleaching by neutralizing the linoleate‐free radical and other free radicals formed in the system. The results of different antioxidants tests shows that BHT, which is the synthetically antioxidant product had the high activity compare to essential oil one.

### EC_50_ values of antioxidant properties

The Table 5 presented the EC_50_ values of antioxidant activities assayed. The antioxidant properties assayed herein are summarized in Table 5. The antioxidant activities with regard to EC_50_ values, b‐carotene method are excellent and in the decreasing order of DPPH method and reducing power with 2.52 mg L^−1^, 2.77 mg L^−1^, and 4.13 mg L^−1^,respectively, for crude oil. The EC_50_ increases with the exposition time. EC_50_ values in DPPH rise to 4.08 mg L^−1^ after 5 days of storage. EC_50_ values in *β*‐carotene rise to 3.11 mg L^−1^ and 5.24 mg L^−1^ after 5 and 10 days storage, respectively. Although BHT exhibited higher antioxidant activity (1.6 mg L^−1^) follow by *β*‐carotene system (2.52 mg L^−1^) and scavenging ability on DPPH radicals (2.77 mg L^−1^).

**Table 5:** EC_50_ values of essential oil of *Bidens pilosa* leaves from various antioxidant properties methods

Antioxidant methods	EC_50_ (mg L^−1^)			
	1	5	10	BHT
DPPH	2.63 < 2.77 > 2.91	3.52 < 4.08 > 4.64	–	1.48 < 1.6 > 1.72
Reducing power	3.71 < 4.13 > 4.55	–	–	3.22 < 3.48 > 3.74
*β*‐carotene	2.31 < 2.52 > 2.73	2.77 < 3.11 > 3.45	4.46 < 5.24 > 6.02	2.02 < 2.13 > 2.24

BHT, Butylated Hydroxytoluene, DPPH, 1‐diphenyl‐2‐picrylhydrazyl.

The EC_50_ value is a widely used parameter to measure the free radical scavenging activity. A lower EC_50_ indicates a higher antioxidant activity (Maisuthisakul et al. 2007). Stanojevic et al. (2009) shown that the EC_50_ values for methanolic extract of *Hieracium pilosella* was higher than those of aqueous and ethanolic extract. A previous study done by Goudoum et al. (2009) shows that the EC_50_ of essential oils of *C. anisata* and *P. glandulosus* depending on the antioxidant method. These results confirm a higher antioxidant activity of essential oil of *B. pilosa*, using the *β*‐carotene system, followed by the scavenging ability on DPPH.

### ET_50_ values of antioxidant properties

With regard to effective time which reduce 50% at which the antioxidant activity was 50% (ET_50_) values of essential oil of *B. pilosa* leaves, antioxidant activities by DPPH radicals are excellent and in decreasing order of *β*‐carotene and reducing power determination (Table 6). The ET_50_ increases with concentration. At 10% of essential oil, the ET_50_ values were 1.74 days and 2.03 days, respectively, for DPPH and *β*‐carotene. ET_50_ values rise to 2.44 days and 2.53 days at the concentration of 15%, respectively, for DPPH and *β*‐carotene. At a concentration of 20%, the ET_50_ of these two methods was 2.88 days. Regarding the reducing power, the ET_50_ (1.59 days) was determined at a concentration of 20%.

**Table 6:** ET_50_ values of essential oil of *Bidens pilosa* leaves from various antioxidant properties methods

Antioxidant methods	ET_50_ (days)		
	10%	15%	20%
DPPH	1.39 < 1.74 > 2.09	1.93 < 2.44 > 2.93	2.77 < 2.88 > 2.89
Reducing power	–	–	1.42 < 1.59 > 1.76
*β*‐carotene	1.75 < 2.03 > 2.31	2.15 < 2.53 > 2.91	2.75 < 2.88 > 2.91

A lower ET_50_ indicates a rapidity of antioxidant activity. The facility with which radicals are formed indicates a greater ability to give the hydrogen of antioxidants (Huang et al. 1995; Huang and Frankel 1997). Tseng et al. (2007) showed that based on the facility of radical formation, large concentrations showed a greater capacity to release the hydrogen. This result confirms the higher antioxidant activity of essential oil of *B. pilosa*, using the power reducing system, followed by the β‐carotene system, and the scavenging ability on DPPH.

## Conclusion

The essential oil *B. pilosa* leaves exhibited different levels of antioxidant activity during two weeks of storage at room condition in all of the models studied. The results from various free radical‐scavenging systems revealed that *B. pilosa* had significant (*P* < 0.05) antioxidant activity and free radical‐scavenging activity. The free radical‐scavenging property mechanisms by which this essential oil is useful when added to foodstuff as well as in traditional medicine. These antioxidant activities although weak compared to the BHT one could be beneficial to consumers of storage products.

## Conflict of Interest

None declared.
